# Morphological Changes in the Placenta of Patients with COVID-19 During Pregnancy

**DOI:** 10.3390/diagnostics15243188

**Published:** 2025-12-13

**Authors:** Kseniia Rudenko, Tatiana Roshchina, Irina Zazerskaya, Dmitry Kudlay, Anna Starshinova, Lubov Mitrofanova

**Affiliations:** 1 Almazov National Medical Research Centre, St. Petersburg 197341, Russia; rudenko_ka@almazovcentre.ru (K.R.);; 2 Department of Pharmacology, Institute of Pharmacy, I.M. Sechenov First Moscow State Medical University, Moscow 119991, Russia; 3 Department of Pharmacognosy and Industrial Pharmacy, Faculty of Fundamental Medicine, Lomonosov Moscow State University, Moscow 119991, Russia; 4 Institute of Immunology FMBA of Russia, Moscow 115522, Russia; 5 Department of Mathematics and Computer Science, St-Petersburg State University, St. Petersburg 199034, Russia

**Keywords:** COVID-19, CD26, morphological changes, placenta, pregnancy, preeclampsia, SARS-CoV-2, VEGF

## Abstract

**Introduction**: The contribution of SARS-CoV-2 infection to the severity of placental alterations in preeclampsia remains unclear. This study aimed to evaluate the morphological changes in placentas of women who experienced COVID-19 during pregnancy, with a focus on the presence or absence of preeclampsia. **Materials and Methods**: The study included placentas from: (1) patients with both COVID-19 during pregnancy and preeclampsia (n = 20, 2022); (2) patients with COVID-19 during pregnancy without preeclampsia (n = 20, 2022); (3) patients with preeclampsia but without COVID-19 (n = 5, 2019); (4) patients with physiological pregnancies without COVID-19 or gestational complications (n = 5, 2019). Histological and immunohistochemical examinations of the placentas were performed using antibodies against the SARS-CoV-2 spike protein, DPP4 (CD26), and VEGF. **Results**: Placentas from patients with both COVID-19 and preeclampsia demonstrated the most pronounced stromal and vascular alterations, including pseudo-infarctions and villous fibrosis. Chorangiosis, excessive fibrinoid deposition in the intervillous space, and accelerated villous maturation with an increased number of syncytial knots were more common in the preeclampsia groups, regardless of prior COVID-19 infection. Symptomatic forms of coronavirus infection were associated with more severe manifestations of malperfusion. Expression of the SARS-CoV-2 spike protein was detected in 78% of syncytiotrophoblast cells and 37% of decidual cells in 28 of 40 placentas from women with previous COVID-19, while its presence in the vascular endothelium, macrophages, and villous fibroblasts was focal, as was CD26 expression. VEGF expression did not differ significantly between patients with and without COVID-19. **Conclusions**: COVID-19 is associated with more pronounced stromal–vascular alterations in the placenta; however, not all of these changes are directly caused by the virus itself but rather reflect the severe course of preeclampsia. Inflammatory alterations are nonspecific for COVID-19, even though CD26 and the SARS-CoV-2 spike protein are detectable in nearly all placental structures, whereas VEGF levels remain comparable to those observed in placentas prior to the coronavirus pandemic.

## 1. Introduction

Despite the official end of the COVID-19 pandemic in 2023, the novel coronavirus infection remains a significant concern in the context of such a vulnerable risk group as pregnant women. According to various meta-analyses and systematic reviews [1,2,3], global rates of maternal mortality and stillbirths increased markedly during the pandemic. Given the continued circulation of new viral variants, COVID-19 still requires further investigation to prevent perinatal complications.

Published data on the association between COVID-19 and gestational complications remain inconsistent, particularly regarding preterm birth [4,5,6] and adverse fetal outcomes [7,8,9]. The prospective INTERCOVID study demonstrated a significant association between COVID-19 and preeclampsia [10]. Moreover, Conde-Agudelo A. et al. [11] reported an increased risk of severe preeclampsia and HELLP syndrome both in symptomatic and asymptomatic cases of coronavirus infection. However, the pathogenesis underlying this association remains unclear.

It is well established that the placenta reflects the pathological effects of infectious agents. Nevertheless, the current literature lacks a consensus regarding the impact of COVID-19 on placental morphology. The most frequently described pathological alterations in placentas from patients with current or past COVID-19 include signs of maternal and fetal vascular malperfusion [12,13,14,15]. According to some authors, SARS-CoV-2 infection may cause coronavirus-induced placentitis, characterized by diffuse tissue destruction, loss of the placenta’s respiratory function, and, consequently, fetal ischemic death [16,17]. However, while some placentas from women with COVID-19 exhibit pronounced morphological abnormalities, others appear indistinguishable from those of healthy mothers [18,19]. Furthermore, the relationship between these morphological changes and preeclampsia, as well as the specific contribution of SARS-CoV-2 infection to the severity of placental pathology, remains uncertain.

The present study aimed to assess morphological alterations in placentas from women who experienced COVID-19 during pregnancy, with particular attention to the presence or absence of preeclampsia.

## 2. Methods

### 2.1. Clinical Characteristics of the Study Groups

A retrospective analysis of archived placental specimens was conducted using materials from the Department of Pathological Anatomy, Almazov National Medical Research Centre (Saint Petersburg, Russia).

The study included four groups of patients:

Group 1—women who had COVID-19 during pregnancy and subsequently developed preeclampsia, who delivered at the Almazov Centre in 2022 (n = 20);

Group 2—women who had COVID-19 during pregnancy without developing preeclampsia, also delivered in 2022 (n = 20);

Group 3—women with preeclampsia but without a history of COVID-19, who delivered before the pandemic in 2019 (n = 5);

Group 4—women with physiological pregnancies without COVID-19 or gestational complications, also delivered in 2019 (n = 5).

The diagnosis of COVID-19 during pregnancy was confirmed by polymerase chain reaction (PCR) detection of SARS-CoV-2 RNA in upper respiratory tract specimens obtained at any stage of gestation.

Among patients with a history of COVID-19, two subgroups were distinguished: asymptomatic (n = 10) and symptomatic (n = 10) infection. Asymptomatic COVID-19 was defined as PCR-confirmed SARS-CoV-2 RNA detection in upper respiratory tract specimens in the absence of any clinical manifestations.

The severity of COVID-19 was determined according to the following criteria:

Mild disease: body temperature < 38 °C; cough, fatigue, sore throat; and absence of criteria for moderate or severe disease.

Moderate disease: body temperature > 38 °C; respiratory rate > 22/min; dyspnea on exertion; CT findings typical of viral pneumonia; SpO_2_ < 95%; and serum CRP > 10 mg/L.

Severe disease: respiratory rate > 30/min; SpO_2_ ≤ 93%; PaO_2_/FiO_2_ ≤ 300 mmHg; altered mental status or agitation; hemodynamic instability (systolic BP < 90 mmHg or diastolic BP < 60 mmHg, urine output < 20 mL/h); CT findings typical of severe viral pneumonia; arterial blood lactate > 2 mmol/L; and qSOFA > 2 points.

Critical disease: persistent high fever; ARDS; respiratory failure requiring ventilatory support (invasive mechanical ventilation); septic shock; multiple organ failure; and CT evidence typical of critical viral lung injury or ARDS.

Moderate preeclampsia was diagnosed based on systolic blood pressure ≥ 140 mmHg and/or diastolic blood pressure ≥ 90 mmHg, proteinuria > 0.3 g/L in two urine samples collected 6 h apart. Severe preeclampsia was diagnosed when systolic blood pressure was ≥160 mmHg and/or diastolic blood pressure was ≥110 mmHg, proteinuria exceeded 5 g/day or 3 g/L in two urine samples collected 6 h apart, and/or at least one sign of organ dysfunction was present, including neurological symptoms, renal insufficiency, impaired liver function, epigastric or right upper quadrant painpulmonary edema, thrombocytopenia, or hemolysis in peripheral blood.

### 2.2. Histological Examination

Histological evaluation of placental tissue was performed using light microscopy after hematoxylin and eosin staining. For each patient included in the study, at least 10 microscopic fields were examined in 10 placental fragments. Microscopic assessment included the evaluation of villous maturation relative to gestational age, signs of maternal and fetal vascular malperfusion, and the localization, extent, and nature of inflammatory infiltrates in all placental structures.

### 2.3. Immunohistochemical Study

Immunohistochemical staining was carried out using the following primary antibodies: SARS-CoV-2 spike protein (rabbit polyclonal antibody, GeneTex, Cat. No. GTX135356; dilution 1:100), VEGF (mouse monoclonal antibody, clone VG1, Thermo Fisher Scientific (Waltham, MA, USA); 1:100), and DPP-4 (CD26; rabbit polyclonal antibody, Thermo Fisher Scientific (Waltham, MA, USA); 1:500).

The mean relative expression density (the proportion of antigen-positive cells of a specific type relative to the total number of those cells per field of view) was calculated by analyzing 10 microscopic fields at ×200 magnification using the Leica LAS Image Analysis System (Leica QWin Plus v3, Leica Microsystems IS, Cambridge, UK) with a Leica DM 4000B microscope.

### 2.4. Statistical Analysis

Statistical analyses were performed using IBM SPSS Statistics version 26.0 (IBM Corp., Armonk, NY, USA) and StatSoft Statistica version 10 (StatSoft Inc., Tulsa, OK, USA). Categorical variables are presented as absolute values and corresponding percentages (%). The Shapiro–Wilk test was used to assess the normality of data distribution. Since the samples did not follow a normal distribution, quantitative data were described as medians (Me) and interquartile ranges (Q1–Q3).

Associations between categorical variables were tested using the chi-squared test. When ≥20% of table cells had expected frequencies < 5 or zero counts, Fisher’s exact test was applied. To determine the significance of differences between groups after overall intergroup comparison (in cases involving more than two groups), a post hoc analysis with Benjamini–Hochberg correction for multiple comparisons was applied to identify statistically significant differences between specific pairs of groups (denoted as p1–2 for differences between Groups 1 and 2. For a limited number of pairwise comparisons of categorical variables, Bonferroni correction was also used. For quantitative variables, intergroup differences were assessed using the Kruskal–Wallis test; pairwise comparisons were performed using the Mann–Whitney U test with Bonferroni correction for multiple testing. A *p*-value < 0.05 was considered statistically significant.

## 3. Results

### 3.1. Clinical Characteristics of the Study Groups

The study groups did not differ significantly in maternal age (*p* = 0.554). The median age in Group 1 (preeclampsia and COVID-19) was 31 years (30; 34); in Group 2 (COVID-19 without preeclampsia)—34.5 years (30; 37); in Group 3 (preeclampsia without COVID-19)—38 years (27.5; 43); and in Group 4 (physiological pregnancy without COVID-19 or gestational complications)—32 years (29.5; 34,5).

Patients did not differ in parity (*p* = 0.806), nor in the prevalence of comorbid conditions, except for chronic arterial hypertension, which was more frequent in the groups with preeclampsia compared with those without it (p1-2, p3-4 = 0.01).

Among patients with preeclampsia and a history of COVID-19 (Group 1), moderate preeclampsia occurred in 3 cases (15%), while severe preeclampsia was observed in 17 cases (85%). In the group with preeclampsia but without COVID-19 (Group 3), moderate preeclampsia was diagnosed in 1 case (20%), and severe preeclampsia in 4 cases (80%).

The timing of delivery was comparable between both preeclampsia groups, with all deliveries being preterm. The median gestational age at delivery in Group 1 (preeclampsia and COVID-19) was 31 weeks (29; 34), and in Group 3 (preeclampsia without COVID-19) it was 29 weeks (25.5; 33.5), with no statistically significant differences (*p* = 0.607). In contrast, the median gestational age in Group 2 (COVID-19 without preeclampsia) was 39 weeks (38; 40), identical to Group 4 (physiological pregnancy without COVID-19 or complications) which was 39 weeks (38; 40), also without statistically significant differences (*p* = 0.944).

Expectedly, statistically significant differences in gestational age at delivery were observed between groups with and without preeclampsia (pairwise comparisons №1–2, №1–4, №2–3): (p1–2 = 0.0006, p1–4 = 0.006, p2–3 = 0.006, respectively).

Selected clinical and anamnestic characteristics of the study participants are presented in Table S1.

Table 1 presents the characteristics of Groups 1 and 2 according to the gestational period and severity of COVID-19.

Unfavorable perinatal outcomes were noted in the group №1 with both COVID-19 and preeclampsia, including one case of antenatal fetal death at 25 weeks of gestation and one case of maternal death in the postpartum period. Additionally, one case of medically induced abortion at 21 weeks and 6 days was recorded in this group due to severe preeclampsia refractory to medical therapy.

According to the assessment of fetal condition in the study groups (Table S2), no statistically significant differences were identified in the frequency of fetal growth restriction, utero-placental or feto-placental Doppler abnormalities, amniotic fluid volume disorders, or fetal distress between the groups with preeclampsia depending on the presence or absence of COVID-19 (Groups 1 and 3), as well as between the groups without preeclampsia (Groups 2 and 4).

Fetal growth restriction and fetal distress during pregnancy occurred more frequently in patients with both preeclampsia and COVID-19 compared with those with COVID-19 but without preeclampsia. In addition, utero-placental or feto-placental Doppler abnormalities were more common in the groups with preeclampsia than in those without it. Neonatal outcomes (Table S3) were also characterized by lower Apgar scores at the first and fifth minutes of life and lower anthropometric parameters in the groups with preeclampsia compared with those without this hypertensive complication; however, COVID-19 itself did not significantly influence these indicators.

### 3.2. Histological Findings

**Figure 1:**
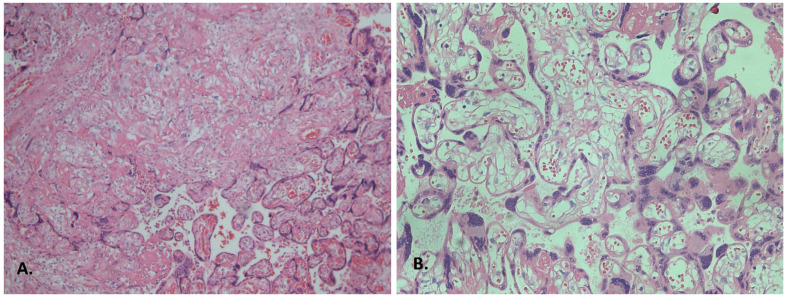
Features of maternal malperfusion in placentas from patients with COVID-19. (**A**) Extensive pseudo-infarction, ×100 magnification; (**B**) Hyperplasia of syncytial knots and distal villous hypoplasia of the chorionic villi. Haematoxylin and eosin staining, ×200 magnification.

Assessment of stromal and vascular disturbances revealed that placentas from patients with preeclampsia following COVID-19 infection exhibited significantly more extensive pseudo-infarctions compared with both the preeclampsia group without COVID-19 and the COVID-19 group without this gestational complication (Table 2, Figure 1A).

In addition, placentas from patients with preeclampsia, regardless of prior COVID-19 infection, demonstrated more pronounced signs of excessive fibrinoid deposition in the intervillous space compared with placentas from women without preeclampsia. Accelerated villous maturation and an increased number of syncytial knots, representing villous dysmorphism, were also more common in preeclampsia groups (Table 2, Figure 1B).

**Figure 2:**
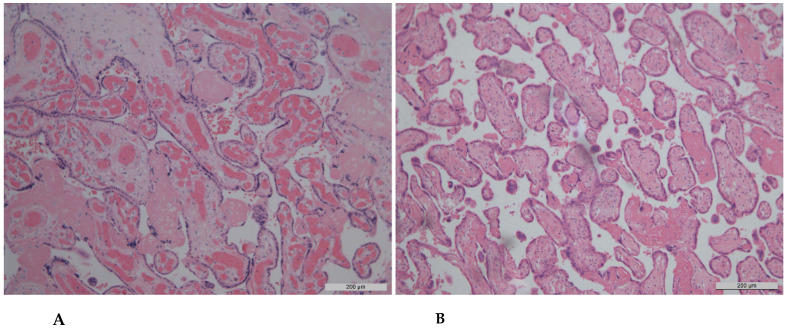
Features of foetal vascular abnormalities in the placenta of a patient with COVID-19 and pre-eclampsia. Haematoxylin and eosin staining, ×200 magnification. (**A**) Chorangiosis of villi with distal hypoplasia; (**B**) Stromal fibrosis and reduced vascularisation of the chorionic villi.

In the context of fetal stromal–vascular disturbances (Table 3), placentas from Group 1 demonstrated the most pronounced karyolysis of vascular wall and villous stromal cells, followed by the development of villous chorion fibrosis (Figure 2B), compared with Groups 2 and 4. However, no statistically significant differences were observed between Groups 1 and 3 (preeclampsia without COVID-19).

Moreover, signs of chorangiosis (Figure 2A) were more frequently detected in placentas from patients with both preeclampsia and COVID-19, with statistically significant differences observed only between Groups 1 and 2. Marked vascularization of stem villi was more common in the preeclampsia groups, regardless of COVID-19 infection status.

#### 3.2.1. Inflammatory Alterations

Evaluation of inflammatory alterations revealed that placentas from patients with both COVID-19 and preeclampsia exhibited more pronounced signs of acute inflammatory response, manifested as chorio-deciduitis, compared with those from patients with COVID-19 without this hypertensive complication. However, no statistically significant differences were found when compared with groups without previous coronavirus infection (Table 4).

Manifestations of chronic inflammation in the form of productive chorio-deciduitis were not statistically significant in intragroup comparisons. Conversely, placentas from patients with preeclampsia, regardless of prior COVID-19 infection, demonstrated more pronounced villitis compared with those from patients without preeclampsia.

**Figure 3:**
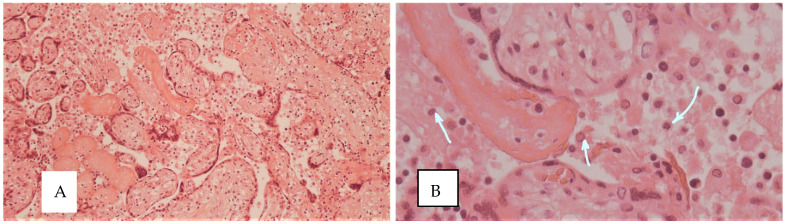
Inflammatory changes in the placenta of a patient from the COVID-19 and preeclampsia group. (**A**) Histiocytic intervillositis, ×200; (**B**) macrophages in the intervillous space (arrows), hematoxylin and eosin staining, ×400.

No pathognomonic or specific inflammatory lesions were identified in the COVID-19 groups. Figure 3A shows histiocytic intervillositis in a patient who contracted COVID-19 during the second trimester, while Figure 3B demonstrates the presence of macrophages in the intervillous space.

#### 3.2.2. The Influence of Clinical Manifestations of COVID-19 on the Severity of Morphological Changes in the Placenta

When assessing the contribution of COVID-19 symptoms to the severity of maternal vascular lesions (Table 5), it was found that delayed maturation of the villous chorion was more pronounced in cases of symptomatic COVID-19. Accelerated maturation of the villous chorion with an increased number of syncytial knots, reflecting villous dysmorphism, was most evident in placentas from patients with preeclampsia, regardless of the presence or absence of clinical manifestations of COVID-19. Extensive pseudo-infarctions were significantly more common in cases of symptomatic COVID-19 and in patients with preeclampsia compared with placentas from patients with asymptomatic SARS-CoV-2 infection. Signs of vascular remodeling in the chorionic plate and major arteries were more characteristic of symptomatic COVID-19, as well as in cases of preeclampsia developing after asymptomatic infection.

#### 3.2.3. Analysis of the Influence of Clinical Manifestations of COVID-19 on the Severity of Fetal Vascular Lesions

Analysis of fetal vascular abnormalities (Table 6) showed that karyolysis of vascular wall and stromal cells, as well as fibrosis of the villous chorion, were more pronounced in placentas from patients with clinical manifestations of COVID-19 and preeclampsia. Complexes of avascular villi were significantly more frequent in placentas from patients with symptomatic COVID-19 and preeclampsia than in the group with asymptomatic COVID-19 without preeclampsia. Signs of chorangiosis were more characteristic of groups with preeclampsia and symptomatic infection, although statistically significant differences were observed only between Groups 1 and 2, and Groups 1 and 4. Stem villus vascularization was more common in preeclampsia groups regardless of the presence of COVID-19 symptoms, although statistically significant differences were found only for groups 1 and 2.

In cases of symptomatic COVID-19 with preeclampsia (Table S4), signs of intra-amniotic infection and purulent choriodeciduitis were observed significantly more often than in the asymptomatic COVID-19 group. Moreover, placentas from patients with symptomatic COVID-19 and from those with preeclampsia showed a significantly higher frequency of deciduitis compared with placentas from patients with asymptomatic COVID-19 without preeclampsia.

### 3.3. Immunohistochemical Analysis of Placental Tissue

**Figure 4:**
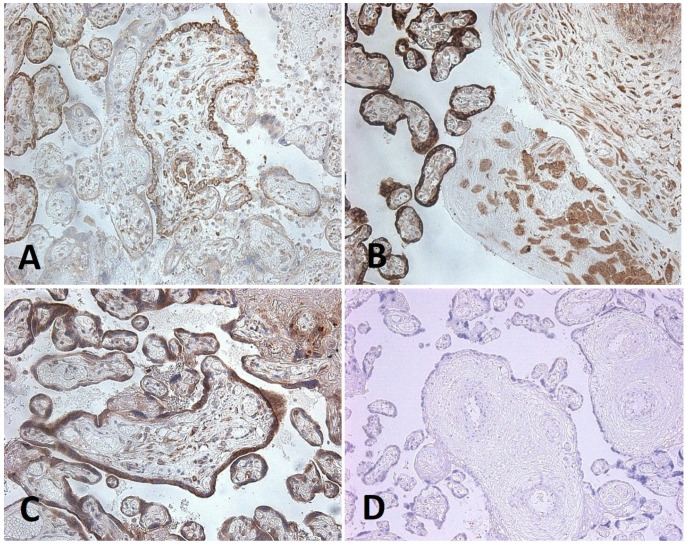
(**A**–**C**)—placenta of a 26-year-old female patient with preeclampsia and a history of COVID-19; SARS-CoV-2 expression (brown staining), ×200. (**A**–**C**)—in the syncytiotrophoblast; (**B**)—in decidual cells and cytotrophoblast; (**C**)—in single endothelial cells, villous macrophages. (**D**)—placenta of a 31-year-old female patient who delivered in 2019, without SARS-CoV-2 expression; ×200.

Immunohistochemical analysis of placentas from groups 1 and 2 revealed pronounced expression of SARS-CoV-2 spike protein in an average of 77.6% of syncytiotrophoblast cells (Table S3, Figure 4). Furthermore, SARS-CoV-2 expression was detected in 37% of decidual cells in 28 of 40 placentas.

Localised expression of the viral antigen was also observed in individual placentas from Groups 1 and 2:

- in the endothelium of villous vessels, detected in five cases, with expression in 50% of endothelial cells in one case;
- in villous macrophages, expression was found in 1% of cells in six cases, 10% in one case, 50% in two cases, and 100% in one case;
- in villous fibroblasts, expression was identified in 30% and 100% of cells in two cases;
- in fibroblasts of the chorionic plate and amnion, expression was present in 50% of cells in two cases and in 100% of cells in another two cases.

**Figure 5:**
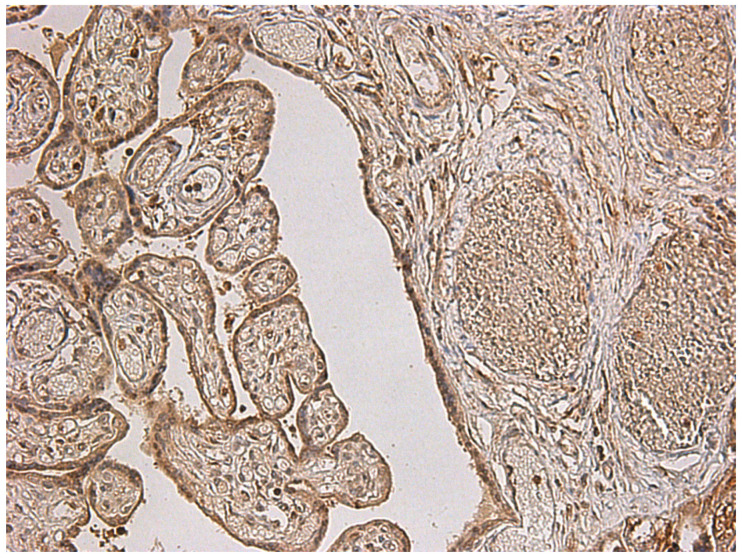
Expression of CD26 (brown staining) in the syncytiotrophoblast and villous macrophages in the placenta of a 26-year-old patient with pre-eclampsia and COVID-19; ×200 magnification.

CD26 expression was observed in the syncytiotrophoblast of placentas in all study groups, including in patients with COVID-19 and preeclampsia (Figure 5). No statistically significant differences were found between groups (Table S5). In placentas from patients with prior SARS-CoV-2 infection, a statistically significant reduction in CD26 expression was observed in villous endothelial cells, villous macrophages, fibroblasts, and decidual cells compared with Groups 3 and 4. Further comparison of CD26 expression density between Groups 1 and 2 demonstrated no significant differences. Thus, following maternal COVID-19 infection, CD26 expression in placental structures was reduced regardless of the presence or absence of pre-eclampsia.

**Figure 6:**
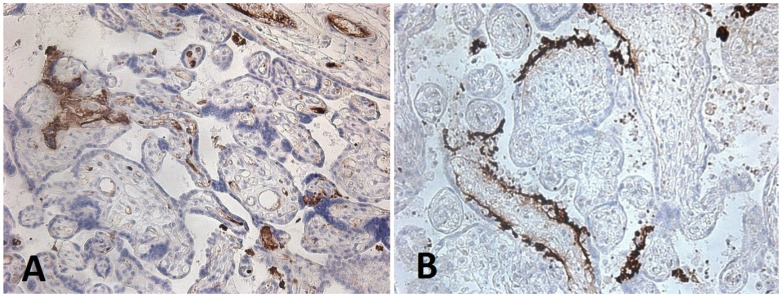
Localized VEGF expression (brown staining) in the placenta of a patient with preeclampsia and COVID-19. (**A**)—in the syncytiotrophoblast and in the endothelium of some villous vessels; (**B**)—expression only in the syncytiotrophoblast; ×200.

Expression of VEGF within placental structures also did not differ between groups and was detected only in individual syncytiotrophoblast cells, endothelial cells of villous vessels, and isolated decidual cells (Figure 6).

## 4. Discussion

The published data do not demonstrate a unified viewpoint regarding the presence of morphological alterations in the placentas of patients who experienced coronavirus infection during pregnancy. According to several authors, SARS-CoV-2 infection is more often associated with signs of maternal and fetal vascular malperfusion [20,21,22], as well as certain inflammatory changes [23], whereas other studies have reported the absence of placental pathology in cases of COVID-19. For instance, in one meta-analysis, no specific changes—particularly vascular or inflammatory—were identified in the placentas of women who had recovered from coronavirus infection [18]. Similar conclusions were reached by Corbetta-Rastelli C.M. et al. (2023) [24]; however, most of their patients had experienced only mild COVID-19. The severity of the infection may represent an important factor, yet data on this issue remain contradictory. The cohort study by Ramey-Collier K. et al. (2022) [25] found no differences in the degree of vascular lesions in the placentas of patients with asymptomatic versus symptomatic COVID-19, including across different trimesters of infection. These controversial results regarding the severity of placental pathology may be related to the fact that studies were conducted during different stages of the COVID-19 pandemic, reflecting shifts in the predominance of specific SARS-CoV-2 variants. Furthermore, it should be noted that most investigations assessed placental morphology in patients with active coronavirus infection at the time of delivery, whereas our study expands current understanding by evaluating the placentas of patients after recovery from COVID-19.

Many studies have demonstrated a significant association between preeclampsia and COVID-19 [26,27]. In this context, it remains important to determine whether COVID-19 itself affects placental morphology, or whether pathological changes develop as a consequence of subsequent gestational complications. Unfortunately, only a few studies have addressed this question. Bachnas M.A. et al. [28] found that pregnancies complicated by either COVID-19 or preeclampsia, as well as by the combination of both, exhibited the most pronounced placental damage related to apoptosis, inflammation, and necrosis. According to the results of our study, placentas from patients with both COVID-19 and preeclampsia demonstrated more severe maternal and fetal vascular abnormalities, such as extensive pseudo-infarctions and villous stromal fibrosis, with prior coronavirus infection contributing significantly to the severity of these lesions. At the same time, the impact of SARS-CoV-2 infection on the degree of fibrinoid deposition in the intervillous space proved insignificant, whereas other studies have identified this as one of the key pathological alterations in placentas of women with COVID-19 [15,21]. Moreover, in our study, the prevalence of certain stromal–vascular abnormalities—such as chorangiosis and accelerated villous maturation with increased syncytial knot formation—did not depend on prior SARS-CoV-2 infection and was equally pronounced in placentas of patients with preeclampsia. Thus, COVID-19 indeed leads to more prominent pathological placental changes; however, not all vascular lesions are directly caused by the virus, and many rather reflect the severe course of preeclampsia.

In our study, symptomatic forms of COVID-19, regardless of the presence of preeclampsia, were associated with more pronounced villous maturation delay, consistent with the findings of other authors [14,21]. Asymptomatic infection less frequently resulted in vascular abnormalities such as extensive pseudo-infarctions, remodeling of chorionic plate vessels, formation of avascular villous complexes, and stem villous hypervascularization, compared with symptomatic cases. These findings confirm the significant contribution of symptom presence and COVID-19 severity to the extent of pathological placental alterations, as also demonstrated by other researchers [29,30].

Furthermore, our data did not reveal any specific inflammatory patterns in the placentas of patients with previous coronavirus infection, which is consistent with the literature [31,32]. Signs of villitis and intervillositis described in some reports of placentas from COVID-19–positive mothers in our study were independent of SARS-CoV-2 infection status and were more commonly observed in patients with preeclampsia. It should be noted that a single case of coronavirus placentitis was recorded at our institution, though it was not included in the present analysis [12]. In that case, inflammatory manifestations did not result in adverse perinatal outcomes—the pregnancy was complicated only by polyhydramnios, and the patient delivered at term without complications. By contrast, Schwartz D.A. et al. (2022, 2023) [16,17] described cases of SARS-CoV-2 placentitis associated with fetal hypoxia and antenatal death. Notably, in most cases, placentitis did not correlate with maternal disease severity, complicating its prediction.

Due to the variable expression of such SARS-CoV-2 mediators in the placenta as the transmembrane receptor ACE2 and TMPRSS2, the placenta represents one of the potential maternal targets during coronavirus infection [33,34]. In our study, SARS-CoV-2 expression was detected in the placenta, being highest in the syncytiotrophoblast and, to a lesser extent, in decidual cells, while in endothelial cells of villous vessels, villous macrophages, fibroblasts, and structures of the chorionic plate and amnion, expression was limited to isolated cells. These results are consistent with previous studies [35,36], which also demonstrated SARS-CoV-2 expression in various placental structures, including the syncytiotrophoblast and decidual cells. Moreover, Radan A.P. et al. (2024) [36] reported that viral replication may persist even after maternal recovery from coronavirus infection, potentially contributing to adverse perinatal outcomes such as intrauterine fetal demise.

The syncytiotrophoblast serves as the principal protective component of the fetoplacental barrier against viral infection, due to its structural characteristics and border localization [37]. Evidently, this structure is the most SARS-CoV-2–tropic, possibly due to high ACE2 expression—particularly in the first trimester—and TMPRSS2 expression peaking in the first and second trimesters [38,39,40]. Dipeptidyl peptidase-4 (DPP4), also known as CD26, is one of the coronavirus-associated receptors and factors (SCARFs), along with basigin (CD147), cathepsin B/L, furin, interferon-induced transmembrane proteins (IFITM1–3), and lymphocyte antigen 6E, all of which may modulate placental permissiveness to SARS-CoV-2 [36]. In our study, decreased CD26 expression was observed in the endothelium of villous vessels, villous macrophages, fibroblasts, and decidual cells, compared with noninfected placentas, which we consider a consequence of prior infection. In placental structures with reduced CD26 expression, SARS-CoV-2 expression was limited to rare cells, whereas in the syncytiotrophoblast—where the median SARS-CoV-2 spike protein expression reached 100%—CD26 expression was also 100%. Thus, the distribution of the viral antigen and its receptor overlapped. According to the review by Tosto V. et al. (2023) [33], CD26 may enhance SARS-CoV-2 infectivity. Reduced activity of circulating DPP4 has been associated with severe COVID-19 and represents a strong prognostic biomarker of mortality [41]. It has been suggested that DPP4 may modify the structure of the SARS-CoV-2 spike protein on the cell membrane, preventing its proteolytic cleavage [42]. Another mechanism underlying reduced CD26 levels may involve virus-induced intracellular alterations interfering with proper DPP4 assembly, thereby decreasing its membrane abundance. DPP4 is expressed in various blood cells, predominantly in T lymphocytes, particularly CD4^+^ T cells [43]. Marked lymphopenia has been well documented in COVID-19 patients [44,45]. Considering these facts, the reduced number of lymphocytes—the main cellular source of soluble DPP4—may explain the low serum DPP4 levels observed in COVID-19 patients. This may also account for the absence of pronounced lymphocytic infiltration in the placentas.

Regarding the expression of vascular endothelial growth factor (VEGF) in SARS-CoV-2–infected placentas, relatively few studies have been published. Shchegolev A.I. et al. [46] reported increased VEGF expression in the syncytiotrophoblast, cytotrophoblast, and endothelial cells of villous vessels, which contrasts with our findings showing no significant differences in VEGF expression between the examined groups. Moreover, due to the lack of differences in VEGF expression between the group with prior COVID-19 without hypertensive complications and the group with both COVID-19 and preeclampsia, our data suggest no direct influence of the virus on placentation or angiogenesis. Nevertheless, further studies are warranted.

In our opinion, the minimal expression of SARS-CoV-2, CD26, and VEGF in the endothelium of villous vessels is noteworthy, since in other organs, endothelial and pericyte infection leads to vascular dysfunction and worsens disease severity [47,48]. Furthermore, viral persistence in these cells has been demonstrated [49]. This likely indicates that the syncytiotrophoblast—where we observed the highest expression of both SARS-CoV-2 and CD26—serves as the primary protective unit of the fetoplacental complex against viral infection.

Thus, inflammatory alterations in the placenta following COVID-19 were nonspecific and did not differ significantly from those observed in uninfected controls. No pathognomonic evidence of coronavirus placentitis or persistent viral infection was detected, in agreement with most previously published studies.

Immunohistochemical analysis revealed predominant SARS-CoV-2 and CD26 (DPP4) expression in the syncytiotrophoblast and decidual cells, whereas endothelial and stromal cells showed minimal expression. The spatial overlap between viral antigen and CD26 expression supports the notion that the syncytiotrophoblast functions as the primary interface and protective barrier against vertical transmission. Reduced CD26 expression in stromal and endothelial compartments may represent a post-infectious effect, whereas the absence of significant changes in VEGF expression suggests a limited direct impact of the virus on placental angiogenesis.

From a broader perspective, these findings underscore the complex interplay between viral infection [50,51,52], maternal vascular adaptation, and placental defense mechanisms. The observed morphological and molecular patterns highlight the resilience of the syncytiotrophoblast as a key structural unit protecting the fetoplacental complex from viral invasion.

Future research should focus on longitudinal and multi-omic studies to elucidate the long-term consequences of maternal SARS-CoV-2 infection on placental function, fetal programming, and neonatal outcomes. Understanding the molecular mechanisms underlying placental susceptibility and compensatory responses to viral injury will be essential for improving maternal-fetal care in the context of emerging infectious diseases.

## 5. Practical Implications

The findings of this study have important practical implications for clinical perinatal care and placental pathology. The demonstrated spectrum of placental lesions associated with SARS-CoV-2 infection—together with the documented patterns of vascular, inflammatory, and immunohistochemical alterations—may assist clinicians and pathologists in better recognizing placental signatures of recent maternal infection. Although antigen detection does not indicate active viral replication, the high prevalence of spike protein in syncytiotrophoblasts highlights the need for careful morphological evaluation in pregnancies complicated by COVID-19.

The observed associations between SARS-CoV-2 infection and features such as maternal vascular malperfusion, fibrinoid deposition, and altered expression of VEGF and CD26 may contribute to improved risk stratification in pregnant patients with current or recent infection. These findings can inform clinical decision-making regarding monitoring strategies, delivery planning, and postnatal evaluation, particularly for pregnancies complicated by hypertensive disorders.

In this publication, the authors aimed to identify possible morphological changes in the placenta of women who contracted SARS-CoV-2 during pregnancy and evaluate the impact of the virus on the fetus and subsequent maternal health [53]. However, the results obtained by the authors differ significantly from our own data due to several limitations such as a small sample size, lack of a control group of healthy pregnant women, and insufficient detailed clinical characteristics for each study participant. We consider it necessary to conduct further large-scale cohort studies to establish specific mechanisms of interaction between the virus and placental tissues and develop effective preventive measures and therapies.

Furthermore, the systematic characterization of placental changes provided by this study establishes a reference framework for future prospective research. Despite the limitations inherent to the retrospective design, the results help to define key histopathological markers that may be relevant for understanding placental susceptibility to viral infections and for refining diagnostic criteria in perinatal pathology.

## 6. Study Limitation

This study has several limitations. Firstly, the sample size of the control groups (Groups 3 and 4) was small, which reduces statistical power, particularly for analyses of VEGF and CD26 expression. Secondly, the use of archived paraffin-embedded placental specimens, collected without a standardized prospective protocol, introduces inherent risks of selection and documentation bias. Although fixation and processing were uniform within the institution, retrospective datasets are unavoidably constrained by variability in clinical documentation.

Thirdly, the retrospective design also limits the ability to incorporate standardized temporal parameters, including controlled assessment of the timing of SARS-CoV-2 infection. While an exploratory comparison of infections by trimester was performed, subgroup sizes remained insufficient to draw definitive conclusions. Importantly, although the retrospective nature of the study does not compromise the validity of the obtained findings, it inherently precludes the possibility of conducting prospective, systematically controlled investigations analogous to those presented here.

Fourthly, the identification of SARS-CoV-2 spike protein by immunohistochemistry must be interpreted with caution. The detection of antigen does not distinguish between active viral replication and residual or phagocytized viral proteins, and the absence of molecular virology assays (qPCR or in situ hybridization) further limits conclusions regarding viral activity in placental tissue.

Finally, although additional correlation analyses were conducted, the inflammatory findings remain nonspecific and did not show strong associations with preeclampsia severity or neonatal outcomes, which should be taken into account when interpreting these results.

## 7. Conclusions

In this study, we systematically evaluated placental histopathology and immunohistochemical expression patterns in pregnancies complicated by SARS-CoV-2 infection, preeclampsia, or their combination. The findings demonstrate that prior COVID-19 is associated with more pronounced maternal and fetal vascular malperfusion, particularly when combined with preeclampsia, whereas several stromal–vascular abnormalities traditionally linked to placental hypoxia were predominantly driven by the severity of preeclampsia itself. Symptomatic SARS-CoV-2 infection was further associated with delayed villous maturation and selected vascular lesions, supporting the contribution of clinical disease severity to placental outcomes.

No specific inflammatory signature attributable solely to SARS-CoV-2 was identified, and inflammatory changes were not consistently distinguishable from those observed in preeclampsia. Immunohistochemistry demonstrated the presence of SARS-CoV-2 spike protein predominantly in the syncytiotrophoblast, alongside parallel expression of CD26, although the interpretation of viral antigen persistence remains limited in the absence of molecular assays. VEGF expression did not differ significantly between groups, suggesting no robust evidence for direct viral effects on placental angiogenesis in the examined cohort.

Taken together, these results indicate that while prior maternal SARS-CoV-2 infection contributes to certain patterns of placental injury, many abnormalities commonly attributed to COVID-19 may instead reflect coexisting gestational complications, particularly preeclampsia. The interplay between infection timing, disease severity, and the placental adaptive response requires further clarification. Future studies with larger placental cohorts, standardized sampling protocols, and integration of molecular virology techniques are needed to more precisely define the pathophysiological impact of SARS-CoV-2 on placental structure and function.

## Figures and Tables

**Table 1:** Characteristics of Groups 1 and 2 according to the timing and severity of COVID-19.

Characteristic	Group 1 (COVID-19 and Preeclampsia (2022), n = 20)		Group 2 (COVID-19 without Preeclampsia (2022), n = 20)	
	n	%	n	%
Trimester of COVID-19 infection				
1st trimester	7	35.0	6	30.0
2nd trimester	10	50.0	12	60.0
3rd trimester	3	15.0	2	10.0
Severity of COVID-19				
Asymptomatic	10	50.0	10	50.0
Mild	8	40.0	9	45.0
Moderate	2	10.0	1	5.0
Severe	0	0	0	0

**Table 2:** Pathological changes in placentas of patients from the studied groups characterizing maternal stromal–vascular alterations.

Pathological Feature	Group 1 (2022): COVID-19 + PE (n = 20)		Group 2 (2022): COVID-19 Without PE (n = 20)		Group 3 (2019): PE Without COVID-19 (n = 5)		Group 4 (2019): No Gestational Complications/COVID-19 (n = 5)		*p*-Value (Fisher’s Exact Test)
	n	%	n	%	n	%	n	%	
Villous maturation discordant with gestational age	4	20.0	1	5.0	2	40.0	0	0	0.128
Delayed villous maturation	4	20.0	2	10.0	1	20.0	0	0	0.651
Dissociated villous maturation	5	25.0	3	15.0	1	20.0	0	0	0.796
Distal villous hypoplasia	4	20.0	2	10.0	1	20.0	0	0	0.651
Accelerated villous maturation and increased syncytial knots	**18**	**90.0**	**10**	**50.0**	**4**	**80.0**	**2**	**40.0**	**0.023** * p_1–2_ = 0.035 *
Excessive fibrinoid deposition in the intervillous space	**13**	**65.0**	**6**	**30.0**	**5**	**100.0**	**1**	**20.0**	**0.006** * p_1–2_ = 0.027 *; p_2–3_ = 0.048 *
Extensive pseudo-infarctions	**20**	**100.0**	**8**	**40.0**	**2**	**40.0**	**0**	**0**	**<0.0001** * p_1–2_ = 0.006 *; p_1–3_ = 0.048 *
Placental infarctions	5	25.0	0	0	0	0	0	0	0.176
—Recent	4	20.0	0	0	0	0	0	0	
—Old	1	5.0	0	0	0	0	0	0	
Intervillous hematomas	6	30.0	1	5.0	0	0	0	0	0.090
Retroplacental hematomas	1	5.0	0	0	0	0	0	0	1.000
Intervillous space narrowing due to hypervillous transformation	4	20.0	8	40.0	3	60.0	0	0	0.106
Remodeling of chorionic plate and main stem arteries	11	55.0	6	30.0	1	20.0	0	0	0.099

Notes: Rows in bold and values marked with * indicate statistically significant differences. Abbreviations: PE—preeclampsia.

**Table 3:** Pathological changes in placentas of patients from the studied groups characterizing fetal stromal–vascular alterations.

Pathological Feature	Group 1 (2022): COVID-19 + PE (n = 20)		Group 2 (2022): COVID-19 Without PE (n = 20)		Group 3 (2019): PE Without COVID-19 (n = 5)		Group 4 (2019): No Gestational Complications/COVID-19 (n = 5)		*p*-Value (Fisher’s Exact Test)
	n	%	n	%	n	%	n	%	
Chorangiosis (≥10 villi with ≥10 vessels each)	14	70.0	5	25.0	2	40.0	2	40.0	0.026 * p_1–2_ = 0.024
Complexes of avascular villi	8	40.0	2	10.0	1	20.0	0	0	0.079
Distorted vascularization of terminal villi	6	30.0	2	10.0	1	20.0	0	0	0.287
Thrombosis of subchorionic space	4	20.0	0	0	1	20.0	0	0	0.130
Karyolysis of vascular wall and villous stromal cells with villous chorion fibrosis	15	75.0	3	15.0	2	40.0	1	20.0	<0.002 * p_1–2_ = <0.002 *; p_1–4_ = 0.024 *
Vascularization of stem villi	11	55.0	4	20.0	3	60.0	0	0	0.023 * p_1–2_ = 0.022 *
Ectasia of villous vessels	6	30.0	6	30.0	2	40.0	2	40.0	1.000
Umbilical vessel abnormalities (dilatation, mural or occlusive thrombosis)	2	10.0	2	10.0	1	20.0	0	0	0.796

Notes: values marked with * indicate statistically significant differences. Abbreviations: PE—preeclampsia.

**Table 4:** Inflammatory changes in placentas of patients from the study groups.

Pathological Changes	Group 1 (2022): COVID-19 and PE (n = 20)	Group 2 (2022): COVID-19 Without PE (n = 20)	Group 3 (2019): PE Without COVID-19 (n = 5)	Group 4 (2019): Without Gestational Complications and COVID-19 (n = 5)	*p* Value (Fisher’s Exact Test)
Signs of intra-amniotic infection	7 (35.0%)	3 (15.0%)	0 (0%)	1 (20.0%)	0.376
Purulent chorio-deciduitis	8 (40.0%)	1 (5.0%)	0 (0%)	1 (20.0%)	0.027 * p_1–2_ = 0.028
Signs of hematogenous infection and chronic inflammatory response					
Productive basal deciduitis	13 (65.0%)	8 (40.0%)	4 (80.0%)	3 (60.0%)	0.306
Productive chorio-deciduitis	9 (45.0%)	3 (15.0%)	3 (60.0%)	0 (0%)	0.001 * p_1–2_ = 0.006
Villitis	8 (40.0%)	1 (5.0%)	2 (20.0%)	0 (0%)	0.019 * p_1–2_ = 0.023; p_2–3_ = 0.023
Intervillositis	1 (5.0%)	0 (0%)	0 (0%)	1 (20.0%)	0.363

Notes: asterisks (*) denote statistically significant differences. PE—preeclampsia.

**Table 5:** Maternal vascular lesions in placentas of patients with COVID-19 and preeclampsia depending on the presence or absence of COVID-19 symptoms.

Pathological Changes	Asymptomatic COVID-19 (2022), n = 10	Asymptomatic COVID-19 and PE (2022), n = 10	Symptomatic COVID-19 (2022), n = 10	Symptomatic COVID-19 and PE (2022), n = 10	*p* Value (Fisher’s Exact Test)
Discrepancy between villous chorion maturity and gestational age	0 (0%)	2 (20%)	1 (10%)	2 (20%)	0.726
Delayed villous chorion maturation	0 (0%)	0 (0%)	2 (20%)	4 (40%)	0.038 *
Dissociated villous chorion maturation	0 (0%)	4 (40%)	3 (30%)	1 (10%)	0.140
Distal villous hypoplasia	2 (20%)	1 (10%)	0 (0%)	3 (30%)	0.463
Accelerated villous chorion maturation and increased syncytial knots	3 (30%)	9 (90%)	7 (70%)	9 (90%)	0.010 * (p_1–2_, p_1–4_ = 0.01)
Excessive fibrinoid deposition in the intervillous space	2 (20%)	5 (50%)	4 (40%)	8 (80%)	0.073
Extensive pseudo-infarctions	1 (10%)	10 (100%)	7 (70%)	10 (100%)	<0.0001 * (p_1–2_, p_1–4_ =0.0006; p_1–3_ = 0.0001)
Placental infarctions—recent—old	0 (0%)	3 (30%)/2 (20%)/1 (10%)	0 (0%)	3 (30%)/2 (20%)/1 (10%)	0.226
Intervillous hematomas	1 (10%)	5 (50%)	0 (0%)	1 (10%)	0.041 **
Retroplacental hematomas	0 (0%)	1 (10%)	0 (0%)	0 (0%)	1.000
Narrowing of the intervillous space due to hypervillosis	3 (30%)	3 (30%)	5 (50%)	1 (10%)	0.329
Remodeling of the chorionic plate and major arterial vessels	0 (0%)	6 (60%)	6 (60%)	5 (50%)	0.010 * (p_1–2_, p_1–3_ = 0.03)

Notes: values marked with * indicate statistically significant differences. Values marked with ** correspond to features with statistically significant differences in the overall comparison, with no statistical differences after post hoc analysis using correction for multiple comparisons. PE—preeclampsia.

**Table 6:** Fetal vascular lesions in placentas of patients with COVID-19 and preeclampsia depending on the presence or absence of COVID-19 symptoms.

Pathological Changes	Asymptomatic COVID-19 (2022), n = 10	Asymptomatic COVID-19 and PE (2022), n = 10	Symptomatic COVID-19 (2022), n = 10	Symptomatic COVID-19 and PE (2022), n = 10	*p* Value (Fisher’s Exact Test)
Chorangiosis (10 villi with 10 vessels)	1 (10.0%)	7 (70.0%)	4 (40.0%)	7 (70.0%)	0.022 (p_1–2_, p_1–4_ = 0.019)
Complexes of avascular villi	0 (0%)	2 (20.0%)	2 (20.0%)	6 (60.0%)	0.017 * (p_1–4_ = 0.036)
Distorted vascularization of terminal villi	0 (0%)	2 (20.0%)	2 (20.0%)	4 (40.0%)	0.207
Thrombosis of the subchorionic space	0 (0%)	2 (20.0%)	0 (0%)	2 (20.0%)	0.300
Karyolysis of vascular wall and stromal cells, villous chorion fibrosis	1 (10.0%)	6 (60.0%)	2 (20.0%)	9 (90.0%)	0.001 * (p_1–4_, p_1–2_, p_3–4_ = 0.001)
Vascularization of stem villi	0 (0%)	6 (60.0%)	4 (40.0%)	5 (50.0%)	0.023 * (p_1–2_ = 0.048)
Ectasia of villous vessels	4 (40.0%)	1 (10.0%)	2 (20.0%)	5 (50.0%)	0.252
Umbilical cord vessels: ectasia, mural thrombi, occlusive thrombi	0 (0%)	2 (20.0%)	2 (20.0%)	0 (0%)	0.300

Notes: values marked with * indicate statistically significant differences. PE—preeclampsia.

## Data Availability

Availability of data and materials. All source data are in the Supplementary Files to the article. If you need clarification or additional information, you can write to the email: rudenko_ka@almazovcentre.ru.

## Supplementary Materials

The following supporting information can be downloaded at: https://www.mdpi.com/article/10.3390/diagnostics15243188/s1, Figure S1, A. SpikeSARS-Cov-2 expression in lung alveolar cells and macrophages (brown staining), ×200. B. Spike SARS-Cov-2 expression in the lung “pre-COVID period” is absent.; Table S1. Selected Clinical and Anamnestic Characteristics of Patients in the Study Groups. Table S2. The condition of the fetus and the frequency of perinatal complications in patients of the study groups, Table S3. Neonatal condition in patients of the study groups, Table S4. Inflammatory changes in the placentas of patients with COVID-19 and preeclampsia depending on the presence or absence of symptoms of coronavirus infection, Table S5. Expression of SARS-CoV-2 Spike Protein, CD26 and VEGF (%) in Placentas of the Studied Groups.

## References

[1] Karimi L., Makvandi S., Vahedian-Azimi A., Sathyapalan T., Sahebkar A. (2021). Effect of COVID-19 on Mortality of Pregnant and Postpartum Women: A Systematic Review and Meta-Analysis. J. Pregnancy.

[2] Chmielewska B., Barratt I., Townsend R., Kalafat E., van der Meulen J., Gurol-Urganci I., O’Brien P., Morris E., Draycott T., Thangaratinam S. (2021). Effects of the COVID-19 pandemic on maternal and perinatal outcomes: A systematic review and meta-analysis. Lancet Glob. Health.

[3] Villar J., Ariff S., Gunier R.B., Thiruvengadam R., Rauch S., Kholin A., Roggero P., Prefumo F., Vale M.S.D., Cardona-Perez J.A. (2021). Maternal and Neonatal Morbidity and Mortality Among Pregnant Women with and Without COVID-19 Infection: The INTERCOVID Multinational Cohort Study. JAMA Pediatr..

[4] Xu K., Sun W., Yang S., Liu T., Hou N. (2024). The impact of COVID-19 infections on pregnancy outcomes in women. BMC Pregnancy Childbirth.

[5] Wei S.Q., Bilodeau-Bertrand M., Liu S., Auger N. (2021). The impact of COVID-19 on pregnancy outcomes: A systematic review and meta-analysis. Can. Med. Assoc. J..

[6] Yao X.D., Zhu L.J., Yin J., Wen J. (2022). Impacts of COVID-19 pandemic on preterm birth: A systematic review and meta-analysis. Public Health.

[7] Rizzo G., Mappa I., Maqina P., Bitsadze V., Khizroeva J., Makatsarya A., D’aNtonio F. (2021). Effect of SARS-CoV-2 infection during the second half of pregnancy on fetal growth and hemodynamics: A prospective study. Acta Obstet. Gynecol. Scand..

[8] Magawa S., Nii M., Enomoto N., Tamaishi Y., Takakura S., Maki S., Ishida M., Osato K., Kondo E., Sakuma H. (2023). COVID-19 during pregnancy could potentially affect placental function. J. Matern. Fetal Neonatal Med..

[9] Smith E.R., Oakley E., Grandner G.W., Ferguson K., Farooq F., Afshar Y., Ahlberg M., Ahmadzia H., Akelo V., Aldrovandi G. (2023). Adverse maternal, fetal, and newborn outcomes among pregnant women with SARS-CoV-2 infection: An individual participant data meta-analysis. BMJ Glob. Health.

[10] Papageorghiou A.T., Deruelle P., Gunier R.B., Rauch S., García-May P.K., Mhatre M., Usman M.A., Abd-Elsalam S., Etuk S., Simmons L.E. (2021). Preeclampsia and COVID-19: Results from the INTERCOVID prospective longitudinal study. Am. J. Obstet. Gynecol..

[11] Conde-Agudelo A., Romero R. (2022). SARS-CoV-2 infection during pregnancy and risk of preeclampsia: A systematic review and meta-analysis. Am. J. Obstet. Gynecol..

[12] Motwani R., Deshmukh V., Kumar A., Kumari C., Raza K., Krishna H. (2022). Pathological involvement of placenta in COVID-19: A systematic review. Infez. Med..

[13] Sharps M.C., Hayes D.J.L., Lee S., Zou Z., Brady C.A., Almoghrabi Y., Kerby A., Tamber K.K., Jones C.J., Waldorf K.M.A. (2020). A structured review of placental morphology and histopathological lesions associated with SARS-CoV-2 infection. Placenta.

[14] Patberg E.T., Adams T., Rekawek P., Vahanian S.A., Akerman M., Hernandez A., Rapkiewicz A.V., Ragolia L., Sicuranza G., Chavez M.R. (2021). Coronavirus disease 2019 infection and placental histopathology in women delivering at term. Am. J. Obstet. Gynecol..

[15] Di Girolamo R., Khalil A., Alameddine S., D’ANgelo E., Galliani C., Matarrelli B., Buca D., Liberati M., Rizzo G., D’ANtonio F. (2021). Placental histopathology after SARS-CoV-2 infection in pregnancy: A systematic review and meta-analysis. Am. J. Obstet. Gynecol. MFM.

[16] Schwartz D.A., Avvad-Portari E., Babál P., Baldewijns M., Blomberg M., Bouachba A., Camacho J., Collardeau-Frachon S., Colson A., Dehaene I. (2022). Placental Tissue Destruction and Insufficiency From COVID-19 Causes Stillbirth and Neonatal Death From Hypoxic-Ischemic Injury. Arch. Pathol. Lab. Med..

[17] Schwartz D.A., Mulkey S.B., Roberts D.J. (2023). SARS-CoV-2 placentitis, stillbirth, and maternal COVID-19 vaccination: Clinical-pathologic correlations. Am. J. Obstet. Gynecol..

[18] Suhren J.T., Meinardus A., Hussein K., Schaumann N. (2022). Meta-analysis on COVID-19-pregnancy-related placental pathologies shows no specific pattern. Placenta.

[19] Corbetta-Rastelli C.M., Altendahl M., Gasper C., Goldstein J.D., Afshar Y., Gaw S.L. (2023). Analysis of placental pathology after COVID-19 by timing and severity of infection. Am. J. Obstet. Gynecol. MFM.

[20] Baergen R.N., Heller D.S. (2020). Placental Pathology in COVID-19 Positive Mothers: Preliminary Findings. Pediatr. Dev. Pathol..

[21] Garg R., Agarwal R., Yadav D., Singh S., Kumar H., Bhardwaj R. (2023). Histopathological Changes in Placenta of Severe Acute Respiratory Syndrome Coronavirus 2 (SARS-CoV-2) Infection and Maternal and Perinatal Outcome in COVID-19. J. Obstet. Gynecol. India.

[22] Gao L., Ren J., Xu L., Ke X., Xiong L., Tian X., Fan C., Yan H., Yuan J. (2021). Placental pathology of the third trimester pregnant women from COVID-19. Diagn. Pathol..

[23] Gesaka S.R., Obimbo M.M., Wanyoro A. (2022). Coronavirus disease 2019 and the placenta: A literature review. Placenta.

[24] Carvajal J., Casanello P., Toso A., Farías M., Carrasco-Negue K., Araujo K., Valero P., Fuenzalida J., Solari C., Sobrevia L. (2023). Functional consequences of SARS-CoV-2 infection in pregnant women, fetoplacental unit, and neonate. Biochim. Biophys. Acta Mol. Basis Dis..

[25] Ramey-Collier K., Craig A.M., Hall A., Weaver K.E., Wheeler S.M., Gilner J.B., Swamy G.K., Hughes B.L., Dotters-Katz S.K. (2022). Symptomatic versus asymptomatic COVID-19: Does it impact placental vasculopathy?. J. Matern. Fetal Neonatal Med..

[26] Di Mascio D., Khalil A., Saccone G., Rizzo G., Buca D., Liberati M., Vecchiet J., Nappi L., Scambia G., Berghella V. (2020). Outcome of coronavirus spectrum infections (SARS, MERS, COVID-19) during pregnancy: A systematic review and meta-analysis. Am. J. Obstet. Gynecol. MFM.

[27] Rao M.G., Toner L.E., Stone J., Iwelumo C.A., Goldberger C., Roser B.J., Shah R., Rattner P., Paul K.S., Stoffels G. (2023). Pregnancy During a Pandemic: A Cohort Study Comparing Adverse Outcomes During and Before the COVID-19 Pandemic. Am. J. Perinatol..

[28] Bachnas M.A., Putri A.O., Rahmi E., Pranabakti R.A., Anggraini N.W.P., Astetri L., Yuliantara E.E., Prabowo W., Respati S.H. Placental damage comparison between preeclampsia with COVID-19, COVID-19, and preeclampsia: Analysis of caspase-3, caspase-1, and TNF-alpha expression. AJOG Glob. Rep..

[29] Flores-Pliego A., Miranda J., Vega-Torreblanca S., Valdespino-Vázquez Y., Helguera-Repetto C., Espejel-Nuñez A., Borboa-Olivares H., Sosa S.E.Y., Mateu-Rogell P., León-Juárez M. (2021). Molecular Insights into the Thrombotic and Microvascular Injury in Placental Endothelium of Women with Mild or Severe COVID-19. Cells.

[30] Meyer J.A., Roman A.S., Limaye M., Grossman T.B., Flaifel A., Vaz M.J., Thomas K.M., Penfield C.A. (2022). Association of SARS-CoV-2 placental histopathology findings with maternal-fetal comorbidities and severity of COVID-19 hypoxia. J. Matern. Fetal Neonatal Med..

[31] Schaumann N., Suhren J.T. (2024). An Update on COVID-19-Associated Placental Pathologies. Ein Update zu COVID-19-assoziierten Pathologien in der Plazenta. Z. Geburtshilfe Neonatol..

[32] Manasova G.S., Stasy Y.A., Kaminsky V.V., Gladchuk I.Z., Nitochko E.A. (2024). Histological and immunohistochemical features of the placenta associated with COVID-19: A systematic review and meta-analysis. Wiad. Lek..

[33] Tosto V., Meyyazhagan A., Alqasem M., Tsibizova V., Di Renzo G.C. (2023). SARS-CoV-2 Footprints in the Placenta: What We Know after Three Years of the Pandemic. J. Pers. Med..

[34] Sayad B., Mohseni Afshar Z., Mansouri F., Salimi M., Miladi R., Rahimi S., Rahimi Z., Shirvani M. (2022). Pregnancy, Preeclampsia, and COVID-19: Susceptibility and Mechanisms: A Review Study. Int. J. Fertil. Steril..

[35] Agostinis C., Toffoli M., Spazzapan M., Balduit A., Zito G., Mangogna A., Zupin L., Salviato T., Maiocchi S., Romano F. (2022). SARS-CoV-2 modulates virus receptor expression in placenta and can induce trophoblast fusion, inflammation and endothelial permeability. Front. Immunol..

[36] Radan A.P., Renz P., Raio L., Villiger A.-S., Haesler V., Trippel M., Surbek D. (2024). SARS-CoV-2 replicates in the placenta after maternal infection during pregnancy. Front. Med..

[37] Kreis N.N., Ritter A., Louwen F., Yuan J. (2020). A Message from the Human Placenta: Structural and Immunomodulatory Defense against SARS-CoV-2. Cells.

[38] Ashary N., Bhide A., Chakraborty P., Colaco S., Mishra A., Chhabria K., Jolly M.K., Modi D. (2020). Single-Cell RNA-seq Identifies Cell Subsets in Human Placenta That Highly Expresses Factors Driving Pathogenesis of SARS-CoV-2. Front. Cell Dev. Biol..

[39] Celewicz A., Celewicz M., Michalczyk M., Woźniakowska-Gondek P., Krejczy K., Misiek M., Rzepka R. (2023). SARS CoV-2 infection as a risk factor of preeclampsia and pre-term birth. An interplay between viral infection, pregnancy-specific immune shift and endothelial dysfunction may lead to negative pregnancy outcomes. Ann. Med..

[40] Cui D., Liu Y., Jiang X., Ding C., Poon L.C., Wang H., Yang H. (2021). Single-cell RNA expression profiling of SARS-CoV-2-related ACE2 and TMPRSS2 in human trophectoderm and placenta. Ultrasound Obstet. Gynecol..

[41] Nádasdi Á., Sinkovits G., Bobek I., Lakatos B., Förhécz Z., Prohászka Z.Z., Réti M., Arató M., Cseh G., Masszi T. (2022). Decreased circulating dipeptidyl peptidase-4 enzyme activity is prognostic for severe outcomes in COVID-19 inpatients. Biomark. Med..

[42] Posadas-Sánchez R., Sánchez-Muñoz F., Guzmán-Martín C.A., Couder A.H.-D., Rojas-Velasco G., Fragoso J.M., Vargas-Alarcón G. (2021). Dipeptidylpeptidase-4 levels and DPP4 gene polymorphisms in patients with COVID-19. Association with disease and with severity. Life Sci..

[43] Boonacker E., Wierenga E., Smits H., Van Noorden C. (2002). CD26/DPPIV signal transduction function, but not proteolytic activity, is directly related to its expression level on human Th1 and Th2 cell lines as detected with living cell cytochemistry. J. Histochem. Cytochem..

[44] Wang F., Nie J., Wang H., Zhao Q., Xiong Y., Deng L., Song S., Ma Z., Mo P., Zhang Y. (2020). Characteristics of peripheral lymphocyte subset alteration in COVID-19 pneumonia. J. Infect. Dis..

[45] Mitrofanova L.B., Makarov I.A., Gorshkov A.N., Runov A.L., Vonsky M.S., Pisareva M.M., Komissarov A.B., Makarova T.A., Li Q., Karonova T.L. (2023). Comparative Study of the Myocardium of Patients from Four COVID-19 Waves. Diagnostics.

[46] Shchegolev A.I., Kulikova G.V., Lyapin V.M., Shmakov R.G., Sukhikh G.T. (2021). The Number of Syncytial Knots and VEGF Expression in Placental Villi in Parturient Woman with COVID-19 Depends on the Disease Severity. Bull. Exp. Biol. Med..

[47] Bernard I., Limonta D., Mahal L.K., Hobman T.C. (2020). Endothelium Infection and Dysregulation by SARS-CoV-2: Evidence and Caveats in COVID-19. Viruses.

[48] Mitrofanova L., Makarov I., Gorshkov A., Vorobeva O., Simonenko M., Starshinova A., Kudlay D., Karonova T. (2023). New Scenarios in Heart Transplantation and Persistency of SARS-CoV-2 (Case Report). Life.

[49] Bussani R., Zentilin L., Correa R., Colliva A., Silvestri F., Zacchigna S., Collesi C., Giacca M. (2023). Persistent SARS-CoV-2 infection in patients seemingly recovered from COVID-19. J. Pathol..

[50] Savchenko A.A., Tikhonova E., Kudryavtsev I., Kudlay D., Korsunsky I., Beleniuk V., Borisov A. (2022). TREC/KREC Levels and T and B Lymphocyte Subpopulations in COVID-19 Patients at Different Stages of the Disease. Viruses.

[51] Kudlay D., Kofiadi I., Khaitov M. (2022). Peculiarities of the T Cell Immune Response in COVID-19. Vaccines.

[52] Gudima G., Kofiadi I., Shilovskiy I., Kudlay D., Khaitov M. (2023). Antiviral Therapy of COVID-19. Int. J. Mol. Sci..

[53] Taş F., Erdemci F., Aşır F., Maraşlı M., Deveci E. (2022). Histopathological examination of the placenta after delivery in pregnant women with COVID-19. J. Health Sci. Med..

