# Supplementary material for: Morphological Changes in the Placenta of Patients with COVID-19 During Pregnancy
Source: Diagnostics (Basel). 2025 Dec 13;15(24):3188. doi: 10.3390/diagnostics15243188 (PMC12731302; doi:10.3390/diagnostics15243188)
Supplement: Supplementary file 1 [file diagnostics-15-03188-s001.zip › diagnostics-3993851-supplementary.pdf]

Sub-Table S1. Selected Clinical and Anamnestic Characteristics of Patients in the Study Groups

Group No.	COVID-19 (yes/no)	Preeclampsia (yes/no)	Age	Parity	Gestational age at delivery	Mode of delivery
1 (2022)	Yes	Yes	34	multiparous	33	emergency caesarean section
1 (2022)	Yes	Yes	36	nulliparous	21	vaginal delivery
1 (2022)	Yes	Yes	29	nulliparous	35	emergency caesarean section
1 (2022)	Yes	Yes	34	multiparous	29	emergency caesarean section
1 (2022)	Yes	Yes	31	nulliparous	29	elective caesarean section
1 (2022)	Yes	Yes	30	nulliparous	31	emergency caesarean section
1 (2022)	Yes	Yes	30	nulliparous	31	emergency caesarean section
1 (2022)	Yes	Yes	31	nulliparous	25	emergency caesarean section
1 (2022)	Yes	Yes	26	nulliparous	25	vaginal delivery
1 (2022)	Yes	Yes	38	multiparous	36	emergency caesarean section
1 (2022)	Yes	Yes	34	multiparous	33	emergency caesarean section
1 (2022)	Yes	Yes	36	multiparous	34	vaginal

						delivery
1 (2022)	Yes	Yes	29	nulliparous	35	emergency caesarean section
1 (2022)	Yes	Yes	34	multiparous	29	emergency caesarean section
1 (2022)	Yes	Yes	31	nulliparous	29	elective caesarean section
1 (2022)	Yes	Yes	30	nulliparous	31	emergency caesarean section
1 (2022)	Yes	Yes	30	nulliparous	31	vaginal delivery
1 (2022)	Yes	Yes	31	nulliparous	25	emergency caesarean section
1 (2022)	Yes	Yes	26	nulliparous	25	vaginal delivery
1 (2022)	Yes	Yes	38	multiparous	36	emergency caesarean section
2 (2022)	Yes	No	30	nulliparous	37	vaginal delivery
2 (2022)	Yes	No	36	nulliparous	40	vaginal delivery
2 (2022)	Yes	No	40	multiparous	39	vaginal delivery
2 (2022)	Yes	No	31	multiparous	38	vaginal delivery
2 (2022)	Yes	No	36	multiparous	40	vaginal delivery
2 (2022)	Yes	No	37	multiparous	40	vaginal delivery

2 (2022)	Yes	No	25	nulliparous	39	elective caesarean section
2 (2022)	Yes	No	38	nulliparous	39	vaginal delivery
2 (2022)	Yes	No	33	multiparous	38	vaginal delivery
2 (2022)	Yes	No	27	nulliparous	41	vaginal delivery
2 (2022)	Yes	No	30	nulliparous	37	vaginal delivery
2 (2022)	Yes	No	36	nulliparous	40	vaginal delivery
2 (2022)	Yes	No	40	multiparous	39	vaginal delivery
2 (2022)	Yes	No	31	multiparous	38	vaginal delivery
2 (2022)	Yes	No	36	multiparous	40	vaginal delivery
2 (2022)	Yes	No	37	multiparous	40	vaginal delivery
2 (2022)	Yes	No	25	nulliparous	39	vaginal delivery
2 (2022)	Yes	No	38	nulliparous	39	vaginal delivery
2 (2022)	Yes	No	33	nulliparous	38	vaginal delivery
2 (2022)	Yes	No	27	nulliparous	41	vaginal delivery
3 (2019)	No	Yes	29	nulliparous	29	emergency caesarean section
3 (2019)	No	Yes	38	multiparous	33	emergency caesarean section

3 (2019)	No	Yes	44	nulliparous	27	emergency caesarean section
3 (2019)	No	Yes	26	multiparous	24	elective caesarean section
3 (2019)	No	Yes	42	multiparous	34	elective caesarean section
4 (2019)	No	No	32	nulliparous	40	vaginal delivery
4 (2019)	No	No	33	multiparous	39	vaginal delivery
4 (2019)	No	No	31	nulliparous	39	vaginal delivery
4 (2019)	No	No	36	nulliparous	37	vaginal delivery
4 (2019)	No	No	28	multiparous	40	vaginal delivery

Notes: vaginal delivery = VD; caesarean section = CS.

Sub Table S2. **The condition of the fetus and the frequency of perinatal complications in patients of the study groups**

Perinatal complications	Group 1 (2022): COVID-19 + PE (n=20)		Group 2 (2022): COVID-19 without PE (n=20)		Group 3 (2019): PE without COVID-19 (n=5)		Group 4 (2019): No gestational complications / COVID-19 (n=5)		p-value (Fisher's exact test)
	n	%	n	%	n	%	n	%	
Small for gestational age fetus	1	5	0	0	0	0	0	0	1.0
Fetal growth restriction	7	35	0	0	2	40	0	0	0.008* p1-2= 0.048*

Utero-placental or fetoplacental Doppler abnormalities		16	80	0	0	3	60	0	0	<0.0001<br>p1-2= 0.0006*<br>p1-4= 0.012*<br>p2-3= 0.024*
Amniotic fluid volume abnormalities	Low water	6	30	0	0	2	40	0	0	0.016**
	Polyhydramnios	4	20	0	0	1	20	0	0	0.130
Fetal distress	During pregnancy	13	65	0	0	2	40	0	0	<0.0001*<br>p1-2= 0.0006*
	During childbirth	0	0	3	15	0	0	1	20.0	0.272
Antenatal fetal death		1	5	0	0	0	0	0	0	1.0
Notes PE – preeclampsia										

Sub-Table S3. Neonatal condition in patients of the study groups

Neonatal parameter	Group 1 (2022): COVID-19 + PE (n=20)	Group 2 (2022): COVID-19 without PE (n=20)	Group 3 (2019): PE without COVID-19 (n=5)	Group 4 (2019): No gestational complications / no COVID-19 (n=5)	*p*-value (Kruskal–Wallis test)
Birth weight, g	1130 (990; 1900)	3365 (3150; 4000)	1400 (575; 1630)	3500 (2925; 3670)	<0.0001*<br>p1–2 = 0.0006*<br>p1–4 = 0.012*<br>p2–3 = 0.006*
Birth length, cm	36 (32; 45)	51.5 (49; 55)	40 (28.5; 42)	52 (50; 52.5)	<0.0001*<br>p1–2 = 0.0006*<br>p1–4 = 0.006*<br>p2–3 = 0.006*<br>p3–4 = 0.048*

Apgar score at 1 minute	6 (5; 7)	8 (8; 8)	7 (6; 7)	8 (8; 8)	<0.0001*<br>p1–2 = 0.0006*<br>p1–4 = 0.006*<br>p2–3 = 0.0006*<br>p3–4 = 0.03*
Apgar score at 5 minutes	7 (7; 8)	9 (9; 9)	8 (7; 8)	9 (9; 9)	<0.0001*<br>p1–2 = 0.0006*<br>p1–4 = 0.0006*<br>p2–3 = 0.0006*<br>p3–4 = 0.03*

Notes

1. Median values with the 25th and 75th percentiles in parentheses are presented as Me (Q1; Q3).
2. Rows in bold and values marked with * indicate statistically significant differences.
3. PE – pre-eclampsia.

Sub Table S4. **Inflammatory changes in the placentas of patients with COVID-19 and preeclampsia depending on the presence or absence of symptoms of coronavirus infection**

Pathological changes	Asymptomatic COVID-19 (2022),<br>n=10		Asymptomatic COVID-19<br>COVID-19 и PE (2022), n=10		COVID-19 symptoms<br>(2022), n=10		COVID-19 symptoms<br>(2022), n=10		p-value (Fisher's exact test)
	abs.	%	abs.	%	abs.	%	abs.	%	
Signs of intra-amniotic infection Suppurative	1	10	0	0	2	20	7	70	0,001*<br>p1–4, p3-4 =0.001
Suppurative choriodeciditis	1	10	0	0	0	0	8	80	<0.0001*<br>p1–4 <0..0001
Signs of hematogenous infection and chronic inflammatory reaction									
Productive basal deciduitis	1	10	7	70	7	70	6	60	0,022*<br>p1–2, p1–3. p1-4= 0.019
Productive choriodeciditis	1	10	4	40	2	20	5	50	0.252

Villuzit	1	10	4	40	0	0	4	40	0.061
Intervillusitis	0	0	0	0	0	0	1	10	1.0
Notes PE – preeclampsia									

Sub Table S5. Expression of SARS-CoV-2 Spike Protein, CD26 and VEGF (%) in Placentas of the Studied Groups

Parameter	Group 1 (COVID-19 and preeclampsia, 2022; n=20)	Group 2 (COVID-19 without preeclampsia, 2022; n=20)	Group 3 (preeclampsia without COVID-19, 2019; n=5)	Group 4 (without COVID-19 and preeclampsia, 2019; n=5)	Kruskal–Wallis test
SARS-CoV-2 expression in syncytiotrophoblast	100 [85; 100]	100 [5; 100]	0 [0; 0]	0 [0; 0]	p = 0.000003
SARS-CoV-2 expression in villous endothelial cells	0 [0; 0]	0 [0; 0]	0 [0; 0]	0 [0; 0]	p = 0.644
SARS-CoV-2 expression in fibroblasts	0 [0; 0]	0 [0; 0]	0 [0; 0]	0 [0; 0]	p = 0.732
SARS-CoV-2 expression in decidual cells	10 [0; 35]	5 [0; 50]	0 [0; 0]	0 [0; 0]	p = 0.012
SARS-CoV-2 expression in villous macrophages	0 [0; 0]	0 [0; 1]	0 [0; 0]	0 [0; 0]	p = 0.276
SARS-CoV-2 expression in the chorionic plate and amnion	0 [0; 0]	0 [0; 0]	0 [0; 0]	0 [0; 0]	p = 0.475
CD26 expression in syncytiotrophoblast	100 [100; 100]	100 [100; 100]	100 [100; 100]	100 [100; 100]	p = 0.555
CD26 expression in villous endothelial cells	0 [0; 20]	0 [0; 100]	100 [100; 100]	100 [100; 100]	p = 0.0003

Parameter	Group 1 (COVID-19 and preeclampsia, 2022; n=20)	Group 2 (COVID-19 without preeclampsia, 2022; n=20)	Group 3 (preeclampsia without COVID-19, 2019; n=5)	Group 4 (without COVID-19 and preeclampsia, 2019; n=5)	Kruskal–Wallis test
CD26 expression in fibroblasts	0 [0; 0]	0 [0; 100]	100 [100; 100]	100 [100; 100]	p = 0.00003
CD26 expression in decidual cells	0 [0; 50]	20 [0; 75]	100 [100; 100]	100 [30; 100]	p = 0.0050
CD26 expression in villous macrophages	0 [0; 0]	0 [0; 40]	100 [100; 100]	100 [100; 100]	p = 0.000003
CD26 expression in the chorionic plate and amnion	0 [0; 0]	0 [0; 50]	100 [100; 100]	100 [100; 100]	p = 0.0002
VEGF expression in syncytiotrophoblast	0.5 [0; 1]	0 [0; 1]	0 [0; 0]	0 [0; 0]	p = 0.467
VEGF expression in villous endothelial cells	1 [1; 16]	1 [0; 3]	0 [0; 0]	1 [0; 1]	p = 0.043
VEGF expression in fibroblasts	0 [0; 0]	0 [0; 0]	0 [0; 0]	0 [0; 0]	p = 0.682
VEGF expression in decidual cells	0 [0; 1]	0 [0; 1]	0 [0; 0]	0 [0; 10]	p = 0.822
VEGF expression in villous macrophages	0 [0; 0.5]	0 [0; 0]	0 [0; 0]	0 [0; 0]	p = 0.306
VEGF expression in the chorionic plate and amnion	0 [0; 0]	0 [0; 0]	0 [0; 0]	0 [0; 0]	p = 0.682

Notes:

1. Data are presented as *Med [Q1; Q3]*.
2. Bolded rows indicate parameters showing statistically significant differences among all four groups simultaneously, adjusted for multiple comparisons using the Bonferroni correction.

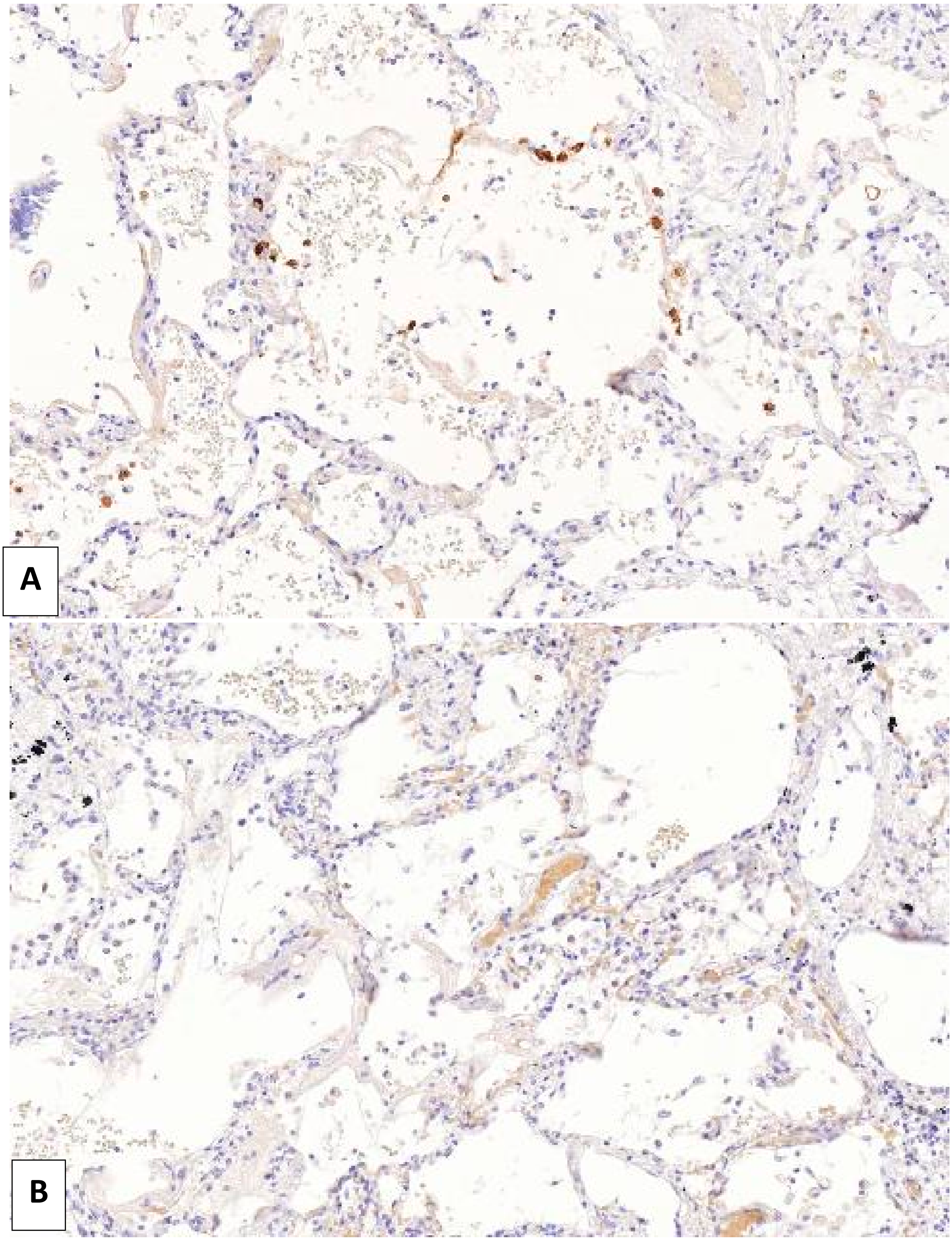

Figure S1 Sup. A. SpikeSARS-Cov2 expression in lung alveolar cells and macrophages (brown staining), x200. B. Spike SARS-Cov 2 expression in the lung "pre-COVID period" is absent.
